# Miltefosine: a novel internal standard approach to lysophospholipid quantitation using LC-MS/MS

**DOI:** 10.1007/s00216-017-0223-z

**Published:** 2017-02-04

**Authors:** A. Ruth Godfrey, Lewis Jones, Mairead Davies, Rachel Townsend

**Affiliations:** Institute of Mass Spectrometry, School of Medicine, Swansea University, Singleton Park, Swansea, SA2 8PP UK

**Keywords:** Lysophosphatidylcholine quantitation, Liquid chromatography-mass spectrometry, Miltefosine, Solid phase extraction, Plasma

## Abstract

Understanding and determining levels of lysophospholipids (LPLs) is of increasing interest to the bioanalytical community as they may be targeted for preparative removal as a matrix interference or as a lead substance as a biomarker of disease. Studies monitoring levels of LPLs have used a range of approaches for quantitation whereby those using an internal standard have used either deuterated analogues of the target LPL or alternative LPLs containing an odd number of carbon atoms within its chain, which can be expensive and difficult to distinguish with other LPLs, respectively. A structural analogue, miltefosine, was investigated as a novel internal standard to quantify a selection of lysophosphatidylcholines (LPCs) of clinical interest. A reverse phase C18 LC-MS/MS method was characterised for 16:0-LPC, 18:1-LPC and 18:0-LPC, showing good sensitivity and linearity for all compounds, with limit of detection (LOD) values <1 μg/mL and *R* ^2^ ≥ 0.97. Quality control (QC) samples were studied to determine accuracy and precision of the method, with values <15% variation for each compound at multiple concentrations. As an example application, we have used this method to detect the amount of LPC breakthrough following solid phase extraction (SPE) of plasma to quantify LPCs as a target species and to remove them as matrix interferences under various conditions typical to clinical work. This study showed that changes in sample pH could adversely affect the capture of the LPCs and their contribution as matrix interferences, with 3.6 μg/mL of 18:1-LPC observed following plasma extraction.

**Electronic supplementary material:**

The online version of this article (doi:10.1007/s00216-017-0223-z) contains supplementary material, which is available to authorized users.

**Graphical Abstract:**
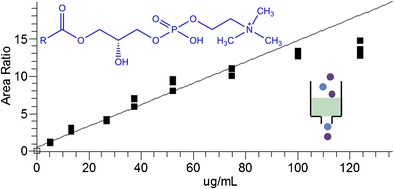
A novel internal standard approach to lysophospholipid quantitation in extracted plasma using miltefosine, with analysis by LC-MS/MS

## Introduction

**Fig. 1:**
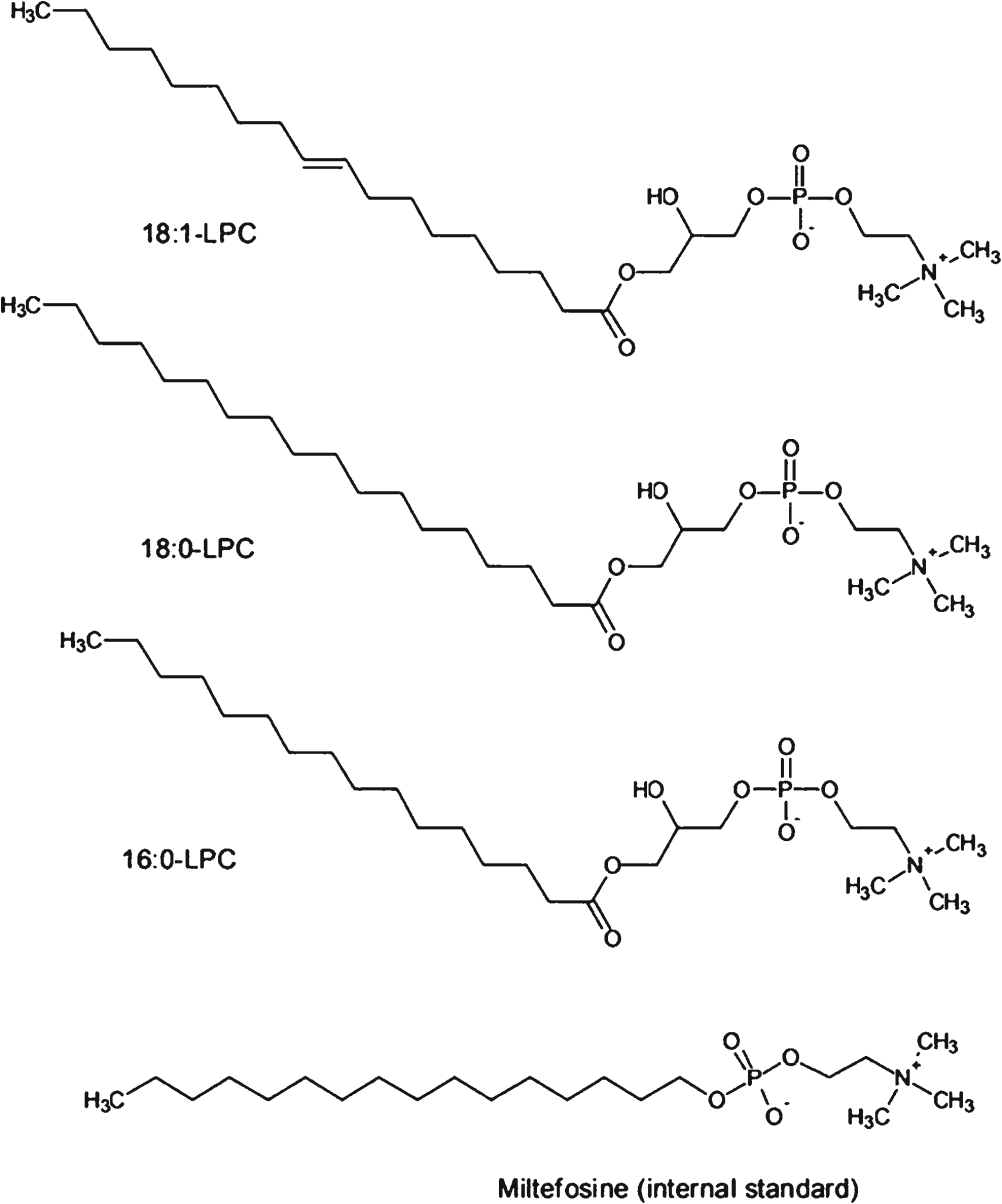
Chemical structure of the lysophosphatidylcholines (1-palmitoyl-sn-glycero-3-phosphocholine (*16:0-LPC*), 1-stearoyl-sn-glycero-3-phosphocholine (*18:0-LPC*), 1-oleoyl-sn-glycero-3-phosphocholine (*18:1-LPC*)) and a structural analogue, miltefosine (internal standard), investigated in this study

Phospholipids (PLs) are the primary component of cell membranes with a characteristic structure and function. Each type of PL consists of a fatty acid tail(s), a linker group (such as glycerol or sphingosine) and a polar head group containing phosphate [1]. Fatty acids can be of a range of carbon chain lengths (e.g. C4-C36) [1] and the polar head group can contain various chemical structures, including choline, ethanolamine and inositol groups. These alternate structures provide great variation in the types of PLs observed in biological samples. Phospholipids are degraded naturally to repair cell membranes using lipolytic enzymes such as phospholipase A; these can remove a single fatty acid forming a lysophospholipid (LPL) of related type (see Fig. 1 for example LPLs). Quantifying levels of PLs and their LPL constituents in biological samples are of increasing interest to the bioanalytical community as these molecules can often interfere with the analytical methods employed for detecting target substances such as pharmaceuticals. Liquid chromatography-mass spectrometry (LC-MS) has become the gold standard method of detection and quantitation of non-volatile analytes at trace levels due to its inherent specificity and sensitivity. For analytes such as pharmaceuticals, atmospheric pressure ionisation (API) methods such as electrospray ionisation (ESI) are often used, especially for analyses in biological samples. Accurate analysis of target analytes within complex biological samples is often difficult without prior preparation or separation of the sample when using this approach as more abundant species, or those that are more amenable to ESI conditions can often mask and interfere with the detection of the target analyte. Phospholipids [2–4] and LPLs [3, 4] are examples of ‘matrix interference’ that can affect the reliable detection of target analytes in biological samples. There are many noted approaches to reduce their impact on the analysis by removal through sample preparation with an extensive review of matrix interference available in the literature [5]. Understanding the levels of lipids, particularly LPLs, is important to estimate the amount of LPL ‘break-through’ when evaluating sample preparation technology and for clinical applications where they may be used as biomarkers of disease, including monitoring lipolytic enzyme activity and as physiological messenger molecules within biochemical signalling pathways.

It is still unclear as to the absolute levels of PLs and LPLs and their various sub-types and this presumably is made more challenging by the interconversion of these lipids that occurs naturally. However, PL concentrations within human plasma have been reported at levels of 1–3 mg/mL [6–8] with those containing a choline head group (i.e. phosphatidylcholine) being the most abundant contributing to approximately 70% [8–10]. Of the glycerophospholipids, the most common chain lengths observed are those containing a saturated C16 or C18 chain and an unsaturated C18 to C20 chain [1]. Therefore, it is understandable why past literature has reported relatively high abundances of lysophosphatidylcholine (∼3.2% of lipid phosphorous detected in plasma) [10] and in particular, a bias in forming those of C16 and C18 chain lengths (i.e. C16:0, C18:0, C18:1, C18:2) in various biological samples [8, 11–13]. However, this landscape of published data has been accumulated using differing methods of detection and quantitation meaning that a range of reported levels remain and could be further influenced by enzyme activity and association with other biomolecules, such as albumin [14]. More recent analytical methods for LPLs have employed hyphenated mass spectrometry techniques and in particular, hydrophilic interaction liquid chromatography- or normal phase liquid chromatography-mass spectrometry (LC-MS) using ESI [15, 16]. However, these conditions may not necessarily be suited to other target analytes (i.e. pharmaceuticals), and prolonged operation using simple conditions typical for reverse phase methods in clinical analyses (water and acetonitrile solvents) have shown evidence of LPC contamination and carryover within the LC system (data acquired in house). The studies monitoring levels of LPCs have used a range of approaches to quantify absolute amounts whereby those adopting an internal standard method have used either deuterated analogues of the target LPC or LPCs containing an odd number of carbon atoms within its chain. Unfortunately, both approaches have limitations and can adversely affect the implementation of these methods. For example, deuterated analogues are often synthesised through bespoke methods resulting in a high purchase cost. Also it is often challenging to maintain the integrity of the deuterated material with the possibility of deuterium exchange under certain experimental conditions [17, 18]. For odd numbered chain LPCs, there are concerns regarding the reliability of assigning the identity of various LPCs for quantitation as there are a number that are isobaric when detected by MS and potentially when using LC too [16]. Given these limitations, there remains a need for an alternative internal standard that may be used for LPC quantitation and is the subject of this paper.

Miltefosine or hexadecyl phosphocholine is a structural analogue of 16:0-LPC without a glycero-linking group between the phosphocholine head group and alkyl chain (see Fig. 1). Unlike deuterated analogues of LPCs, miltefosine is available at relatively low cost and has a similar hydrophobicity (logP) to common LPCs (e.g. 16:0-LPC, 18:0-LPC, 18:1-LPC). Admittedly, this compound is a therapeutic and may not have the uniqueness of deuterated material; however, it is administered specifically for the treatment of the parasitic disease, leishmaniasis [19], within developing countries and is unlikely to be in the majority of samples for clinical study. A validated reverse phase LC-MS method is available for miltefosine, but this is concerned with determinations as the target compound rather than using it as an internal standard for LPC quantitation [19]. To the best of the authors’ knowledge, the latter has yet to be investigated and published. To show the usability of this approach, we have evaluated miltefosine for quantifying a range of LPCs of clinical interest using an LC-MS/MS method compatible in detecting both LPCs as the target analytes and a selection of example pharmaceuticals which may be subject to matrix interference from the presence of LPCs. For proof of application within a clinical scenario, we have also used this method to detect the absolute amounts of LPCs in control plasma matrix which have been carried through a solid phase extraction (SPE) procedure typically used for clinical and pharmaceutical methods.

## Experimental

### Instrumentation

Sample separation was carried out using a Surveyor autosampler and an MSPumpPlus (Thermo Fisher Scientific, Runcorn, UK) LC system on a reverse phase Thermo Fisher Scientific Hypersil Gold LC column (1.0 × 100 mm, 5 μm) operated with a Hypersil guard cartridge, suitable for general separations. Mass spectrometry detection was performed using an LCQ ion trap (Thermo Fisher Scientific, Hemel Hempstead, UK) operated using an ESI source in positive mode. Instrument control and data analysis were carried out using Xcalibur 2.0.7 software with data processed using both the Quan Browser Xcalibur tool and Microsoft Excel.

### Chemicals and materials

All standard reference materials, 1-palmitoyl-*sn*-glycero-3-phosphocholine (16:0-LPC), 1-stearoyl-*sn*-glycero-3-phosphocholine (18:0-LPC), 1-oleoyl-*sn*-glycero-3-phosphocholine (18:1-LPC), miltefosine, propranolol, diphenhydramine and loratadine, had a purity of ≥99%, with the exception of miltefosine, which was ≥98% and were purchased from Sigma-Aldrich (Poole, UK). Solid phase extraction (SPE) cartridges containing C18 sorbent were provided by Porvair Sciences (Wrexham, UK). Plasma matrix containing EDTA anticoagulant was obtained from Seralab (West Sussex, UK) with solvents, methanol and water (both HPLC grade) from Fisher Scientific (Loughborough, UK). Ammonium solution (10% in water) was sourced from Sigma-Aldrich (Poole, UK) and formic acid from Fisher Scientific (Loughborough, UK); these were used for pH modification of the LC mobile phase and for sample modification for SPE.

### Stock solutions, calibration standards and quality control samples

Due to the limited amount of LPC standard material, stock solutions were prepared at 1 mg/1.5 mL concentrations in a 50:50 mixture of solvents used for the LC mobile phase (10 mM ammonia in 70:30 methanol/water (A), and 10 mM ammonia in methanol (B)). Calibration standards were made from the stock solutions as a mixture in 50:50 mobile phase solution containing LPCs and the internal standard (IS), miltefosine, at concentrations ranging 5.3–124.0 μg/mL (16:0-LPC), 5.3–78.7 μg/mL (18:0-LPC), 5.3–52.0 μg/mL (18:1-LPC), with each standard containing 6.7 μg/mL of miltefosine. Standard blanks (a ‘double blank’ and an ‘internal standard blank’, *S*_0_) were also included within the quantitative evaluation to assess the presence of carryover and contamination during sample preparation. The internal standard blank, *S*_0_, was also used to determine the limit of detection (LOD) for the method. For method evaluation for quantitation, quality control samples (QCs) were prepared in the same fashion as the calibration standards at a low, mid and high concentration within the calibration range (i.e., 37.3, 74.7, 124.0 μg/mL (16:0-LPC), 32.0, 53.3, 78.7 μg/mL (18:0-LPC), 26.7, 36.0, 52.0 μg/mL (18:1-LPC)) with each containing 6.7 μg/mL of miltefosine.

### Solid phase extraction (SPE)

For initial proof of principle of LPL capture and break-through for a typical clinical application, a Microlute C18 30 mg, 1.5F frit 96-well plate was tested under different pH conditions (acid (0.1% *v*/*v* formic acid), base (0.1% *v*/*v* ammonia) and neutral) using solvent samples spiked with LPC and an example complex clinical matrix, plasma. Solvent (water) samples were spiked at appropriate concentrations of LPCs at the upper end of the expected concentration range for plasma (i.e. 16:0-LPC 117.3, 18:0-LPC 53.3, 18:1-LPC 32.0 μg/mL). The spiked solvent sample or plasma was dispensed as a 125 μL volume onto the cartridge along with 375 μL of methanol (with or without pH modifier) to encourage protein precipitation prior to elution. After application, the sample mixture was eluted under positive pressure, collecting the eluent for concentration and solvent exchange via evaporation. Dried extracts were reconstituted using the 50:50 mobile phase mixture containing miltefosine internal standard ready for analysis by LC-MS. Break-through of LPC and performance of the C18 SPE cartridge were determined using the percentage recovery of LPC in the extract. A matrix effect measurement was also determined to assess the variation in signal for individual LPCs and miltefosine caused by matrix interference from the co-extractives (including other LPCs).

### Liquid chromatography conditions

Mobile phases consisted of 10 mM ammonia (pH 10) in 70:30 methanol/water (A) and 10 mM ammonia in methanol (B). Reverse phase chromatography was carried out using the Hypersil Gold column operated with a guard cartridge of the same material using these solvents as a gradient programme at 50 μL/minute. Starting conditions consisted of 10% B and were held for 2 min following injection of the sample. The solvent gradient was increased from 10% B to 70% B over 7.5 min and then to 100% B until 17.5 min. The column was washed in 100% B over 5 min and returned to 10% B over 1 min. The column was conditioned at the starting solvent mixture of 10% B for 13 min ready for the next injection. Samples were injected as 5 μL volumes using a partial loop injection mode and kept at 4 °C while on the autosampler tray. A wash (and flush) solvent of 50:50 A/B mobile phase was used for the needle, syringe and loop at a volume of 750 μL for each injection.

### Mass spectrometry conditions

Data was acquired online using ESI operating in positive ion mode, at 4.5 kV with a capillary temperature and voltage of 200 °C and 14 V, respectively. A sheath gas flow of 60 (arbitrary units) was used to aid desolvation in ESI. Data was acquired as a full mass scan over a mass range of *m/z* 100–750, product ion scan (for determining fragment ions where appropriate) and selected reaction monitoring (SRM) for quantitation. Final SRM transitions can be found in Table 2.

### Data processing

Mass spectrometry data was processed using Xcalibur 2.0.7 using the ISIS algorithm and Quan Browser tool to obtain peak area and other relevant statistics. Further data processing not possible in Quan Browser was undertaken manually using Microsoft Excel 2007. Quality control (QC) samples were used with the calibration standards to evaluate the quantitative ability of the method tested. For typical figures of merit for quantitation (i.e. accuracy, precision and LOD), the following equations and definitions were used:

$$ \begin{array}{l}\mathrm{Accuracy}=\frac{\mathrm{calculated}\kern0.5em \mathrm{concentration}-\mathrm{true}\kern0.5em \mathrm{concentration}}{\mathrm{true}\kern0.5em \mathrm{concentration}}\times 100\hfill \\ {}\mathrm{Precision}=\frac{\mathrm{standard}\kern0.5em \mathrm{deviation}\kern0.5em \mathrm{of}\kern0.5em \mathrm{calculated}\kern0.5em \mathrm{concentration}}{\mathrm{mean}\kern0.5em \mathrm{calculated}\kern0.5em \mathrm{concentration}}\times 100\hfill \\ {}\mathrm{LOD}=3\times \mathrm{Standard}\kern0.5em \mathrm{deviation}\kern0.5em \mathrm{of}\kern0.5em \mathrm{the}\kern0.5em \mathrm{concentration}\kern0.5em \mathrm{of}\kern0.5em \mathrm{the}\kern0.5em \mathrm{blank}\kern0.5em \mathrm{sample}\hfill \end{array} $$

All other equations (and calculations) used will be described in the relevant sections with references.

## Results and discussion

### Analyte identification by mass spectrometry

Each LPC species and the internal standard, miltefosine, were identified according to the *m/z* corresponding to the monoisotopic mass of the protonated molecules; 496.5, 522.5, 524.5 and 408.5 were observed as primary ion species for 16:0-LPC, 18:1-LPC, 18:0-LPC and miltefosine, respectively (see Table 2). Product ion scans obtained from previous in-house work for the precursor molecule ions of LPCs showed primary fragment ions attributable to a loss of 18 mass units, generating ions at *m/z* 478.0, 504.0 and 506.0 for 16:0-LPC, 18:1-LPC and 18:0-LPC, respectively, presumably from a loss of water from a hydroxyl group within the glycerol section of the structure. Miltefosine however did not show this loss of water but a primary fragment ion also typical with LPCs at *m/z* 184, consistent with the phosphatidylcholine head group. These product ions were chosen to test the quantitative method using selected reaction monitoring (SRM) as shown in Table 2.

### Characterisation and optimisation of liquid chromatography method

**Fig. 2:**
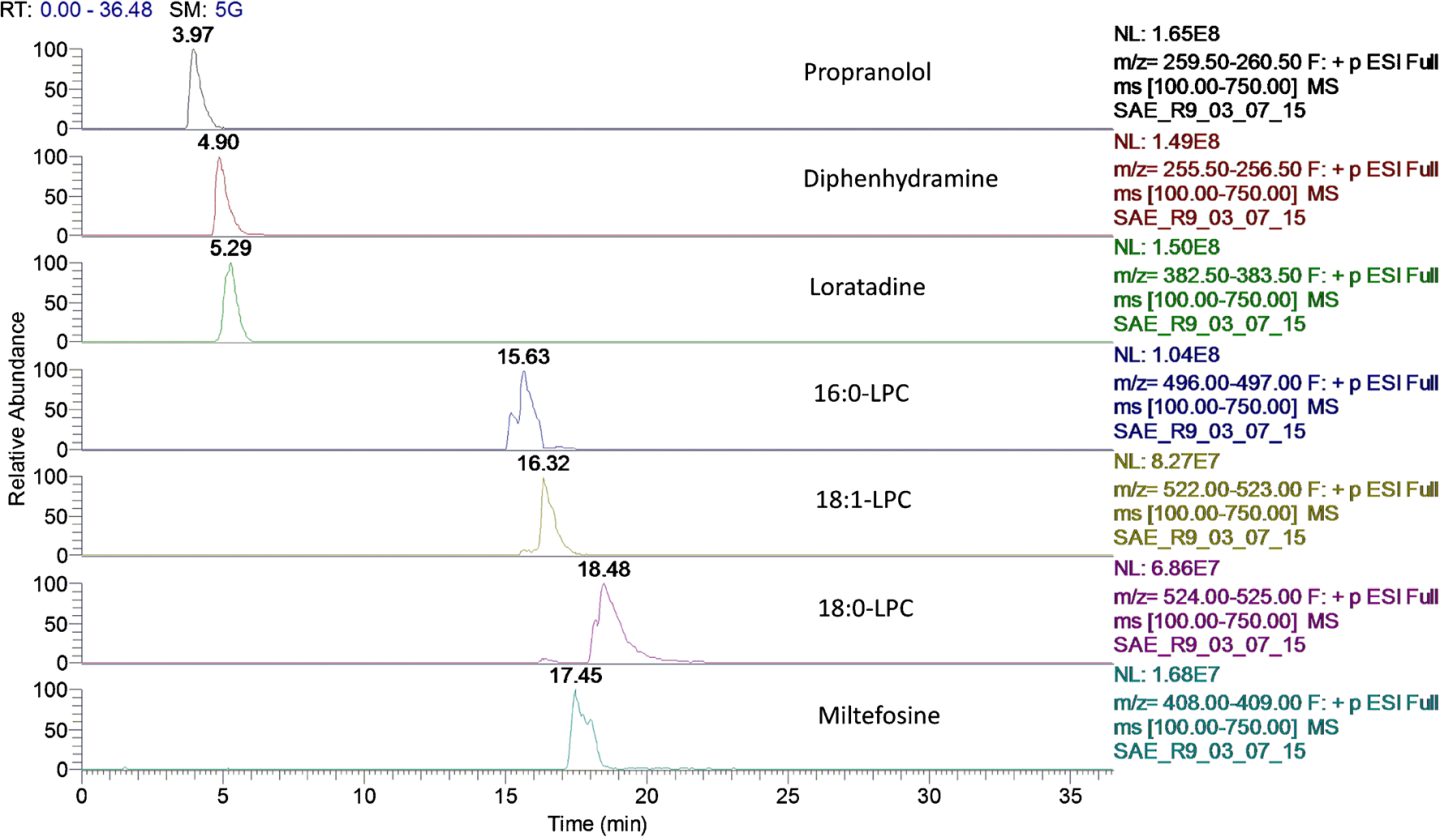
Extracted ion chromatograms of example pharmaceuticals, 16:0-LPC, 18:1-LPC and 18:0-LPC and miltefosine (internal standard)

Typical methods for separating LPCs consist of water and acetonitrile mixtures [4, 18]. Unfortunately, following repeated application of high concentration standards to evaluate the chromatography, carryover was observed, particularly for the 18:0-LPC species. Given this, alternative injection wash volumes and solvents were evaluated but little improvement was observed. An alkali (pH10) water/methanol mobile phase method was investigated [19] showing near baseline separation with a relatively broad solvent gradient and no evidence of carryover. This solvent system was optimised to increase sample throughput yet maintain the chromatographic separation; with these chromatographic conditions, the column wash stage could be shortened to 5 min without experiencing carryover further decreasing the analysis time for each sample. For the envisaged clinical application, the initial gradient conditions were kept at a low elution strength to accommodate more polar target species, whereby this method could be used as a multi-residue approach for LPCs and more polar chemistries. This was tested using a selection of three common pharmaceuticals as example target analytes (propranolol, diphenhydramine and loratadine) and these chromatographic conditions proved suitable for the analysis showing baseline separation and good peak shape (see Fig. 2).

The chromatographic behaviour of the mobile phase system was evaluated to ensure the separation of the compounds was maintained over several days of operation. Chromatography was monitored by injecting a sample repeatedly on two different days; this showed changes in retention time and relative retention per day of %CV <0.92% on day 1 and <2.72% on day 2. To describe the reproducibility of analyte retention, an F-test calculation was used to compare data on day 1 and day 2. After 3 days, the mean retention time did appear to increase and this was confirmed following the application of an appropriate F-test. However, as this was observed for all analytes, it had little impact on the separation and the method was investigated further (without modification) regarding its potential for quantitation.

As expected for reverse phase column chemistry, the analyte retention coincided with lipophilicity (log P) with 18:0-LPC showing the longest retention time consistent with a greater expected interaction on the column. Miltefosine is structurally similar to LPCs but does not contain a glycero-connecting group consistent within LPCs. This considerably affects the calculated log P of the structure (=3.58); however, this could be advantageous with miltefosine likely to elute within the range of the LPCs chosen for this work. Upon further study, this was confirmed showing a retention time between 18:1- and 18:0-LPC. Details of lipophilicity and chromatographic separation (retention times) can be found in Table 1. With the chromatographic method, all the pharmaceuticals eluted at relatively early retention times as expected.

**Table 1:** Physiochemical information and chromatographic data for each lysophosphatidylcholine (16:0-LPC, 18:1-LPC, 18:0-LPC) and the internal standard, miltefosine (*N* = 10 for day 1, 5 for day 2)

Compound	Molecular formula	MW	Log P	Mean retention time (minutes)		Mean adjusted retention time (minutes)		Retention time precision (minutes)		Adjusted retention time precision (minutes)	
				Day 1	Day 2	Day 1	Day 2	Day 1	Day 2	Day 1	Day 2
16:0-LPC	C_24_H_50_NO_7_P	495.63	2.84	15.59	16.78	14.46	15.45	0.46	2.23	0.51	2.54
18:1-LPC	C_26_H_52_NO_7_P	521.67	3.38	16.30	17.51	15.18	16.18	0.58	2.31	0.68	2.63
18:0-LPC	C_26_H_54_NO_7_P	523.68	3.90	18.44	19.66	17.32	18.33	0.59	1.94	0.63	2.19
Miltefosine (IS)	C_21_H_46_NO_4_P	407.57	3.58	17.44	19.02	16.32	17.70	0.76	2.42	0.92	2.72

### Method evaluation for quantitation

To ensure that the chromatographic peak observed is representative of the amount injected on column, it is essential that a sufficient number of mass spectra are recorded during the relevant elution (retention) time for the analyte of interest. Typically, this will be in the order of 10–15 data points (mass spectra) per chromatographic peak. To achieve this, a balance is often sought between mass scan parameters (i.e. the speed of acquisition, whereby SRM scans are generally less time consuming) and chromatographic separation. Hence, where possible, quantitative methods will typically employ an SRM approach to achieve sufficient accuracy of chromatographic peak shape; this with added gains in sensitivity from this scan type enable sensitive and selective quantitation with a good degree of accuracy. As the number of target analytes increases, the number of SRM scans will also increase. When performed sequentially, this can limit the number of relevant data points achieved during the chromatographic peak for the target SRM. An approach to overcome this is to segment the acquisition method, only recording those scans specific to the analytes eluting at relevant times (in this case a full mass scan for screening of analyte transformation products and relevant SRM(s) for quantitation). However, as previously mentioned, this is reliant on stable chromatographic conditions to capture the LC peak. Limited sampling points (mass spectra) during chromatographic method development highlighted that this segmented approach would be necessary as a multi-residue method. When applied, this segmented acquisition, recording the screening full mass scan and the SRMs for the relevant analyte class (i.e. pharmaceuticals or LPCs) was deemed suitable, achieving at least 15 data points across the chromatographic peak.

### Injection repeatability and reproducibility of miltefosine for use as an internal standard

The aim of an internal standard is to normalise for any changes in ionisation during the analysis and is particularly important for techniques that are prone to interference from other analytes present in complex samples. Chromatography can help alleviate this matrix interference but may not completely remove it, and for applications such as quantitation, accounting for this effect is key to accurate measurement. However, isotopically labelled analytes are often very expensive and previously used structural analogues of LPC can be difficult to distinguish from other LPCs, particularly if they are not resolved by the chromatographic method [16]. Miltefosine is an alternative structural analogue, showing sufficient differences in mass for selectivity but a similar lipophilicity to the target LPCs of this study (see Table 1). The repeatability and reproducibility of the miltefosine signal was monitored by injecting a sample repeatedly on two different days. The SRM chromatogram was integrated to obtain the chromatographic peak area for each run and this showed little change per day with a %CV 5.56% on day 1 and 9.39% on day 2. Given the limited fluctuation in the ionisation of miltefosine under these chromatographic conditions (and an appropriate retention time), this was tested further for the quantitation of the LPCs as a possible internal standard. This was monitored throughout the quantitative work with the LPCs as peak area ratio (please see Table S4 and S5 of Electronic Supplementary Material (ESM)).

### Selectivity

Blank samples (both a ‘double blank’ and a blank containing internal standard only, *S*_0_) were used judiciously throughout the batch to detect the presence of carryover and ensure the selectivity of the method. These samples were made from the same matrix as the calibration standards and quality control samples (QCs) to ensure their accuracy. Unlike some of the earlier method development work with traditional reverse phase acetonitrile-based LC methods, there was good selectivity using the chosen SRM transitions and no evidence of carryover at the relevant retention times.

### Statistical testing for homoscedasticity of calibration data

To ensure that correct regression statistics are used to accurately describe the relationship between response (or relative response to the internal standard) and concentration of the target analyte, this should be tested with regards to homoscedasticity (or variance) within the concentration range used. Often analytical techniques can show a greater degree of variability in response (and therefore calculated concentration) at the extremes of the values tested. This can affect the validity and accuracy of the calibration graph in determining concentration, particularly at low concentrations, and an appropriate regression model in establishing this observed relationship should be used. The calibration data acquired with this method was processed by integrating the SRM chromatographic peak for several standards of increasing concentration of each LPC. These were injected in triplicate with a calibration set before and after the QC samples to account for any changes in performance of the method during the batch. Each standard included a constant amount of the internal standard, miltefosine, to normalise the LPC response. The calibration range was based on approximate levels believed to be present in a typical clinical (plasma or serum) sample and the proposed estimated proportions described in previous publications [6–12]. Therefore, each LPC had different calibration ranges to account for these estimated proportions (see ‘Experimental’ section and Table 2 for more details). All LPC peak areas were normalised using the integrated peak area of miltefosine to generate a relative response factor (RRF) for the calibration graph. Using linear regression statistics, initial data showed that linearity would be considerably improved with the omission of the top standard (S8) as this standard showed evidence of signal saturation for all species. In the spirit of good practice, this standard was excluded from all further data analysis and any study (unknown) samples of future analyses that are detected at this concentration should be diluted for detection within the linear range.

**Table 2:** Mass spectral data acquisition information and quantitative data (dynamic range, linearity represented by the coefficient of determination, *R* ^2^, limit of detection (LOD), mean accuracy and precision) for each lysophosphatidylcholine (16:0-LPC, 18:1-LPC, 18:0-LPC) and the internal standard, miltefosine (*N* = 5)

Compound	Precursor ion (*m/z*)	Fragment ion (*m/z*)	SRM transition (*m/z*)	Dynamic range (μg/mL)	Linearity (*R* ^2^)	LOD (μg/mL)	Mean Accuracy (%)		Precision (%)	
							QC1	QC2	QC1	QC2
16:0-LPC	496.5	478.0, 184.0	496.5–478.0	5.3–100.0	0.9698	0.15	7.34	−8.03	9.34	4.09
18:1-LPC	522.5	504.0, 184.0	522.5–504.0	5.3–44.0	0.9783	0.06	−5.66	−11.14	8.19	3.57
18:0-LPC	524.5	506.0, 184.0	524.5–506.0	5.3–62.7	0.9819	0.40	5.24	−3.84	10.20	4.60
Miltefosine (IS)	408.5	184.0, 125.0	408.5–184.0	–	–	–	–	–	–	–

The adjusted calibration range was tested and evaluated in terms of homoscedasticity (i.e. assessing the suitability of equal weighting regression statistics, *w*_*i*_ = 1) according to the methods set out in Almeida et al. [20]. This was undertaken using two methods: comparing a plot of the residual *y*-values versus concentration, and testing statistical differences of the variances of the lowest and highest standard in the calibration range by performing an F-test. The initial results showed high calculated F-values that exceeded the F-test statistic indicating that the data was heteroscedastic for two of the LPCs, 16:0-LPC and 18:0-LPC, with values of 21.65 and 38.51, respectively. Given these results, the data was further tested and compared with 1/*x* and 1/*x* ^2^ weighting regression factors (*w*_*i*_ = 1/*x* and *w*_*i*_ = 1/*x* ^2^) to establish if there was a more appropriate model to determine concentration. The calculated percentage relative error (%RE) of the back-calculated concentrations was determined for the calibration standards. This was tested by either comparing this value versus concentration in the plot described above (whereby values should reside as close to the *x*-axis as possible, see ESM Figs. S1-S3) or by determining the total %RE, with the most appropriate weighting factor having the lowest %RE. It proved prudent to evaluate these weighting factors as the 1/*x* regression model did provide the lowest %RE for the data set at −0.038, −0.036 and −0.002% for 16:0-LPC, 18:1-LPC and 18:0-LPC, respectively (see ESM Tables S7-S9 for more information) and this was chosen for quantifying the target LPCs.

### Linearity

Using a weighted function of 1/*x*, improved regression statistics were observed. All LPCs now showed a correlation coefficient, (*R*) >0.98, and a coefficient of determination, (*R* ^2^) ≥0.97, for a dynamic range of 5.3–100.0, 5.3–44.0 and 5.3–62.7 μg/mL for 16:0-LPC, 18:1-LPC and 18:0-LPC, respectively. These statistics indicate a good correlation for the regression models describing the relative response of the LPCs versus concentration (individual regression statistics are included in Table 2 and the ESM Table S10 for further information).

### Accuracy

The accuracy of the calibration method to quantify levels of each LPC was tested using QC samples at concentrations within the established dynamic range (see ‘Experimental’ section and Table 2 for details). These were analysed and the relative responses inputted into the regression equation to determine concentration. The difference between the calculated concentration and the ‘true’ concentration (that was spiked) was used to establish the accuracy of the quantitation. For determining the level of ‘acceptable’ accuracy, criteria relevant for clinical and pharmaceutical industries was followed as guidance [21]; all QC samples should be within 15% of the true concentration except at the limit of quantitation where it may not exceed 20% accuracy. Given the ‘high’ QC sample of the original set now exceeded the adjusted calibration range of the regression equation, it was subsequently omitted from further data analysis. Of the remaining QC samples, a good level of accuracy was observed with all five replicate samples for each of the LPC compounds showing values less than the threshold percentage. Subsequently, the mean accuracy values for each LPC showed acceptable accuracy and are shown in Table 2 with additional data present within the ESM. These results are encouraging, indicating that this method is capable of quantifying levels of each LPC using miltefosine as an internal standard to a good degree of accuracy.

### Precision

This was established using the relative standard deviation (%RSD) of the calculated concentrations for each QC level for the LPCs (see Table 2). The results showed good precision with both QC levels meeting the guidance criteria of 20 and 15% for low and mid QC concentration levels [21], respectively, for all LPCs. As expected, the poorest precision was observed at the lowest concentration QC (limit of quantitation); however, this did not exceed 10% for the three LPCs. This data shows that reliable measures of concentration may be achieved using this method within the tested concentration range.

### Sensitivity

Sensitivity of the LC-MS method may be described through several methods; the slope of the calibration graph or by statistically or empirically describing other figures of merit, such as the limit of detection (LOD) using QC samples. The LOD is considered as the ‘smallest measure…that can be detected with reasonable certainty for a given analytical procedure’ [22]. Given that the regression statistics appear to be heteroscedastic for a linear unweighted relationship, it was deemed that transposing the standard error of the regression (*S*_*y*/*x*_) in place of the standard deviation of the blank for the equation in the data processing section would be inappropriate, and therefore the statistical method often employed unsuitable [23]. Instead, the response of three replicate injections of a standard blank containing internal standard (*S*_0_) was used to calculate the LOD giving rise to concentrations of 0.15, 0.06 and 0.40 μg/mL for 16:0-LPC, 18:1-LPC and 18:0-LPC, respectively. These values are in reasonable agreement with the signal/noise of the first standard in the concentration range showing average values above 100:1 for each LPC.

### Applicability of established method

Lysophospholipids are considerable matrix interferences when analysing other substances in clinical samples such as plasma. Solvent samples spiked with LPCs and a pooled plasma sample were extracted using the C18 solid phase cartridges under various pH conditions (acid, base and neutral) to test LPC breakthrough and matrix interference. This approach was chosen as a similar method had been the subject of phospholipid removal for the extraction of clinical samples and the detection of pharmaceuticals as the target substances [24], and it may provide the opportunity to quantify LPCs as target molecules, and how these may be removed as a matrix interference. Acid, base and neutral elution conditions are commonly employed in clinical applications to segregate compound chemistries; however, this can often result in variations in analyte/interference recovery and observed matrix effects. For example, despite the anticipated strong interaction of the LPCs on the C18 sorbent, pH modification may alter retention by subtly changing their chemistry (i.e. groups that are partially dissociated), potentially leading to breakthrough. This approach may also show the applicability of the LC-MS method described to detect and quantify LPCs in a scenario that is of interest to clinical work. Spiked solvent samples/plasma and the crash solvent (methanol with or without pH modification) were drawn through the column and the resulting extract evaporated to dryness. Each condition was tested in triplicate with extracts reconstituted in 50:50 A/B mobile phase mixture containing an appropriate amount of the internal standard, miltefosine, to quantify any LPC detected. To confirm method selectivity for the internal standard within the sample, ‘blanks’ were also extracted with no discernible signal detected above the LOD. With the solvent sample extraction, less than 4% recovery (break-through) was observed for each LPC under acid, base and neutral conditions (see Table 3). Limited matrix effects were observed for each LPC and miltefosine, with values typically within 94–102, 83–88 and 100–108% for acid, base and neutral conditions, respectively. Within plasma extracts 16:0-LPC was observed above the LOD for both acidic and basic extractions at 18.8 and 0.3 μg/mL, respectively, indicating the SPE sorbent ability to capture endogenous LPC had been compromised if we consider the estimated concentrations of total LPC in plasma (i.e. 3.2% [10] of 1–3 mg/mL [6–8]). Given the relative abundance estimations of the LPCs from previous literature, it was anticipated that 16:0-LPC would be most likely to be observed if the sorbent became saturated, followed by 18:0-LPC and 18:1-LPC. While 18:0-LPC was also observed under acidic conditions at a calculated concentration of 3.6 μg/mL, 18:1-LPC appeared to remain on the column with extract levels below the detection limit of the method. Overall acidic extractions did show a greater chance of LPC breakthrough and this should be considered when performing extractions under these conditions for other target substances. However, the data did indicate that the extraction efficiency can be quite varied with only some replicates showing breakthrough; a possible cause of this may be differences in efficiency of protein crash following pH modification and competition on the SPE sorbent for LPC retention.

**Table 3:** Mean percentage matrix effects and recovery of LPCs and miltefosine calculated using spiked solvent samples following C18 SPE with or without pH modification (*N* = 3)

Compound	Mean matrix effects and recovery of LPC detected in spiked solvent extract (%)					
	Acid (0.1% formic acid)		Base (0.1% ammonia)		Neutral	
	Matrix effects	Recovery	Matrix effects	Recovery	Matrix effects	Recovery
16:0-LPC	97.06	0.81	83.18	0.66	101.04	3.14
18:1-LPC	94.70	0.34	83.67	0.35	104.67	0.69
18:0-LPC	94.50	0.16	84.97	0.15	100.38	0.11
Miltefosine (IS)	102.74	0.14	87.88	0.09	108.00	0.14

LPLs are of interest to the clinical community both as a problematic class of biomolecules that can behave as a matrix interference for analyses of clinical samples by LC-MS/MS and as target messenger molecules that have the potential to be used as biomarkers. Therefore there is a developing need to quantify these lipids to ensure appropriate clean-up methods are in place when analysing other target species by LC-MS and for monitoring disease state. Current analytical LC-MS methods have limitations including carryover of lysophospholipids, and limited selectivity and high cost of internal standards chosen for quantitative work. Here we have developed and characterised a novel approach to LPL quantitation using several lysophosphatidylcholine (LPC) species common to clinical samples as the test molecules. This method employs a more cost effective, novel internal standard, a structural analogue of LPC, miltefosine; to the best of the authors’ knowledge, this has not been published in this form or for this application before. The analytical method has been evaluated in terms of chromatographic stability and performance for quantifying the selected LPCs. Data acquired has shown a good level of sensitivity (within that required for monitoring estimated clinical LPC levels), accuracy and precision for quantitation. It has also been successfully applied to a simple plasma extraction using SPE under a range of conditions to detect the level of LPC breakthrough for a common clinical scenario, providing an example of target LPC quantitation and removal as a matrix interference. These results suggest that miltefosine has the potential to be used as a viable alternative internal standard for determining LPC concentrations and warrants future testing in more challenging clinical applications.

## Electronic supplementary material

Below is the link to the electronic supplementary material.

ESM 1 (PDF 428 kb)
