# Supplementary material for: Miltefosine: a novel internal standard approach to lysophospholipid quantitation using LC-MS/MS
Source: Anal Bioanal Chem. 2017 Feb 4;409(11):2791–800. doi: 10.1007/s00216-017-0223-z (PMC5366175; doi:10.1007/s00216-017-0223-z)
Supplement: Supplementary file 1 — (PDF 428 kb) [file 216_2017_223_MOESM1_ESM.pdf]

Analytical and Bioanalytical Chemistry

Electronic Supplementary Material

Miltefosine: a novel internal standard approach to lysophospholipid quantitation using LC-MS/MS

A. Ruth Godfrey, Lewis Jones, Mairead Davies, Rachel Townsend

Table S1 Chromatographic repeatability data for day 1 of the lysophosphatidylcholines (LPCs) and the internal standard miltefosine

Day 1										
**Compound *(m/z)***	**Run**	**Solvent front (minutes)**	**Retention time, RT (minutes)**	**Adjusted RT (minutes)**	**Mean RT**	**Mean adjusted RT**	**Standard Deviation RT**	**Standard Deviation adjusted RT**	**%CV RT**	**%CV adjusted RT**
**Miltefosine (408.5)**	….r1	1.13	17.44	16.31	17.44	16.32	0.1324	0.1497	0.76	0.92
	….r2	1.17	17.41	16.24						
	….r3	1.13	17.41	16.28						
	….r4	1.09	17.41	16.32						
	….r5	1.09	17.69	16.60						
	….r6	1.13	17.29	16.16						
	….r7	1.13	17.29	16.16						
	….r8	1.13	17.44	16.31						
	….r9	1.13	17.45	16.32						
	….r10	1.13	17.46	16.33						
**16:0-LPC (496.5)**	….r1	1.13	15.57	14.44	15.59	14.46	0.0712	0.0732	0.46	0.51
	….r2	1.17	15.66	14.49						
	….r3	1.13	15.51	14.38						
	….r4	1.09	15.51	14.42						
	….r5	1.09	15.71	14.62						
	….r6	1.13	15.57	14.44						
	….r7	1.13	15.51	14.38						
	….r8	1.13	15.54	14.41						
	….r9	1.13	15.63	14.50						
	….r10	1.13	15.64	14.51						
**18:1-LPC (522.5)**	….r1	1.13	16.38	15.25	16.30	15.18	0.0941	0.1038	0.58	0.68
	….r2	1.17	16.29	15.12						
	….r3	1.13	16.18	15.05						
	….r4	1.09	16.27	15.18						
	….r5	1.09	16.48	15.39						
	….r6	1.13	16.20	15.07						
	….r7	1.13	16.24	15.11						
	….r8	1.13	16.26	15.13						
	….r9	1.13	16.32	15.19						
	….r10	1.13	16.40	15.27						
**18:0-LPC (524.5)**	….r1	1.13	18.43	17.30	18.44	17.32	0.1086	0.1093	0.59	0.63
	….r2	1.17	18.49	17.32						
	….r3	1.13	18.50	17.37						
	….r4	1.09	18.31	17.22						
	….r5	1.09	18.58	17.49						
	….r6	1.13	18.36	17.23						
	….r7	1.13	18.23	17.10						
	….r8	1.13	18.52	17.39						
	….r9	1.13	18.48	17.35						
	….r10	1.13	18.51	17.38						

Table S2 Chromatographic repeatability data for day 2 of the lysophosphatidylcholines (LPCs) and the internal standard miltefosine

Day 2										
**Compound (*m/z*)**	**Run**	**Solvent front (minutes)**	**Retention time, RT (minutes)**	**Adjusted RT (minutes)**	**Mean RT**	**Mean adjusted RT**	**Standard Deviation RT**	**Standard Deviation adjusted RT**	**%CV RT**	**%CV adjusted RT**
**Miltefosine (408.5)**	….r1	1.30	19.07	17.77	19.02	17.70	0.4605	0.4808	2.42	2.72
	….r2	1.30	19.77	18.47						
	….r3	1.35	18.97	17.67						
	….r4	1.35	18.57	17.27						
	….r5	1.34	18.74	17.44						
**16:0-LPC (496.5)**	….r1	1.30	16.97	15.67	16.78	15.45	0.3735	0.3927	2.23	2.54
	….r2	1.30	17.25	15.95						
	….r3	1.35	16.83	15.53						
	….r4	1.35	16.56	15.26						
	….r5	1.34	16.28	14.98						
**18:1-LPC (522.5)**	….r1	1.30	17.74	16.44	17.51	16.18	0.4052	0.4252	2.31	2.63
	….r2	1.30	18.02	16.72						
	….r3	1.35	17.57	16.27						
	….r4	1.35	17.21	15.91						
	….r5	1.34	17.01	15.71						
**18:0-LPC (524.5)**	….r1	1.30	19.91	18.61	19.66	18.33	0.3816	0.4014	1.94	2.19
	….r2	1.30	20.11	18.81						
	….r3	1.35	19.68	18.38						
	….r4	1.35	19.47	18.17						
	….r5	1.34	19.13	17.83						

Table S3 Chromatographic reproducibility data (to 2dp) for lysophosphatidylcholines (LPCs) and the internal standard miltefosine. Reproducibility is determined using an F-test with n=10 for day 1 and n=5 for day 2. For F (9,4) the critical value derived from statistical tables = 3.63

**Compound (*m/z*)**	**F-calculated value retention time**	**F-calculated value adjusted retention time**
**Miltefosine (408.5)**	12.09	9.46
**16:0-LPC (496.5)**	27.50	25.99
**18:1-LPC (522.5)**	18.52	15.22
**18:0-LPC (524.5)**	12.35	12.20

Table S4 Repeatability of the miltefosine signal for day 1 represented by the precision of the peak areas obtained over multiple chromatographic runs and different days

Compound (*m/z*)	Run	Peak Area	Relative Response Factor (RRF)	Mean Peak Area	Standard Deviation Peak Area	Mean RRF	Standard Deviation RRF	%CV Peak Area	%CV RRF
**Miltefosine (408.5)**	….r1	105139254.24		104193375.98	5789651.234			5.56	
	….r2	113353485.62							
	….r3	105470470.56							
	….r4	104562445.22							
	….r5	106425959.78							
	….r6	111018390.97							
	….r7	97977686.86							
	….r8	104414097.26							
	….r9	99535840.53							
	….r10	94036128.79							
**16:0-LPC (496.5)**	….r1	1585965640.25	15.08	1600096636.75	67020835.35	15.37	0.34	4.19	2.19
	….r2	1683013059.28	14.85						
	….r3	1605890861.99	15.23						
	….r4	1619823107.53	15.49						
	….r5	1662821643.15	15.62						
	….r6	1650726727.84	14.87						
	….r7	1529551167.27	15.61						
	….r8	1633385052.46	15.64						
	….r9	1568872337.12	15.76						
	….r10	1460916770.64	15.54						
**18:1-LPC (522.5)**	….r1	1321168895.51	12.57	1344221073.62	51956538.79	12.92	0.40	3.87	3.08
	….r2	1403012366.68	12.38						
	….r3	1364647564.40	12.94						
	….r4	1399854798.30	13.39						
	….r5	1399517218.04	13.15						
	….r6	1364928512.72	12.29						
	….r7	1274990318.81	13.01						
	….r8	1336400853.14	12.80						
	….r9	1322916019.86	13.29						
	….r10	1254774188.79	13.34						
**18:0-LPC (524.5)**	….r1	1947761525.22	18.53	2011731923.37	58382979.48	19.34	0.70	2.90	3.64
	….r2	2065692315.72	18.22						
	….r3	2025446850.40	19.20						
	….r4	2012378788.86	19.25						
	….r5	2078219137.69	19.53						
	….r6	2085014345.01	18.78						
	….r7	1963630386.33	20.04						
	….r8	2018586930.15	19.33						
	….r9	2015255661.05	20.25						
	….r10	1905333293.25	20.26						

Table S5 Repeatability of the miltefosine signal for day 2 represented by the precision of the peak areas obtained over multiple chromatographic runs and different days

Compound (*m/z*)	Run	Peak Area	Relative Response Factor (RRF)	Mean Peak Area	Standard Deviation Peak Area	Mean RRF	Standard Deviation RRF	%CV Peak Area	%CV RRF
**Miltefosine (408.5)**	….r1	99243260.83		87095775.54	8181979.65			9.39	
	….r2	91378410.12							
	….r3	84429264.35							
	….r4	80058950.94							
	….r5	80368991.46							
**16:0-LPC (496.5)**	….r1	1447880384.27	14.59	1320104035.50	98103091.36	15.18	0.40	7.43	2.64
	….r2	1400629846.50	15.33						
	….r3	1272264565.91	15.07						
	….r4	1256042295.82	15.69						
	….r5	1223703085.02	15.23						
**18:1-LPC (522.5)**	….r1	1238639870.23	12.48	1113018462.18	93708383.48	12.79	0.24	8.42	1.85
	….r2	1181045316.81	12.92						
	….r3	1082253815.63	12.82						
	….r4	1047461454.18	13.08						
	….r5	1015691854.04	12.64						
**18:0-LPC (524.5)**	….r1	1857166523.59	18.71	1712649237.68	111477074.56	19.71	0.65	6.51	3.30
	….r2	1808042943.16	19.79						
	….r3	1646222232.49	19.50						
	….r4	1614451811.63	20.17						
	….r5	1637362677.55	20.37						

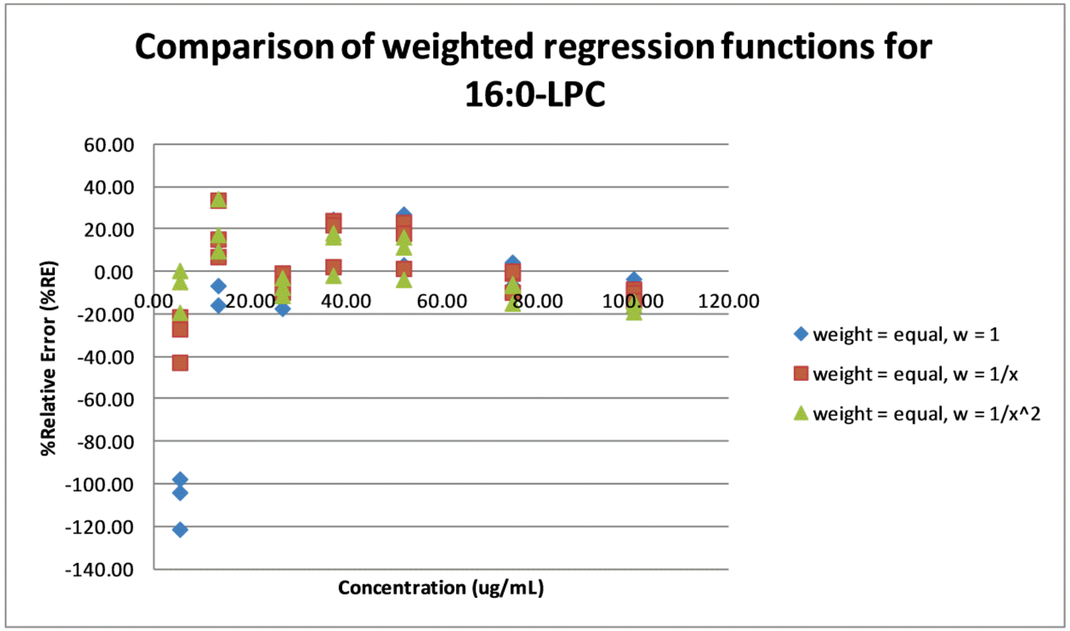

Fig. S1 Plot of the residual y-values versus concentration for 16:0-LPC at unweighted and 1/x weighted regression functions

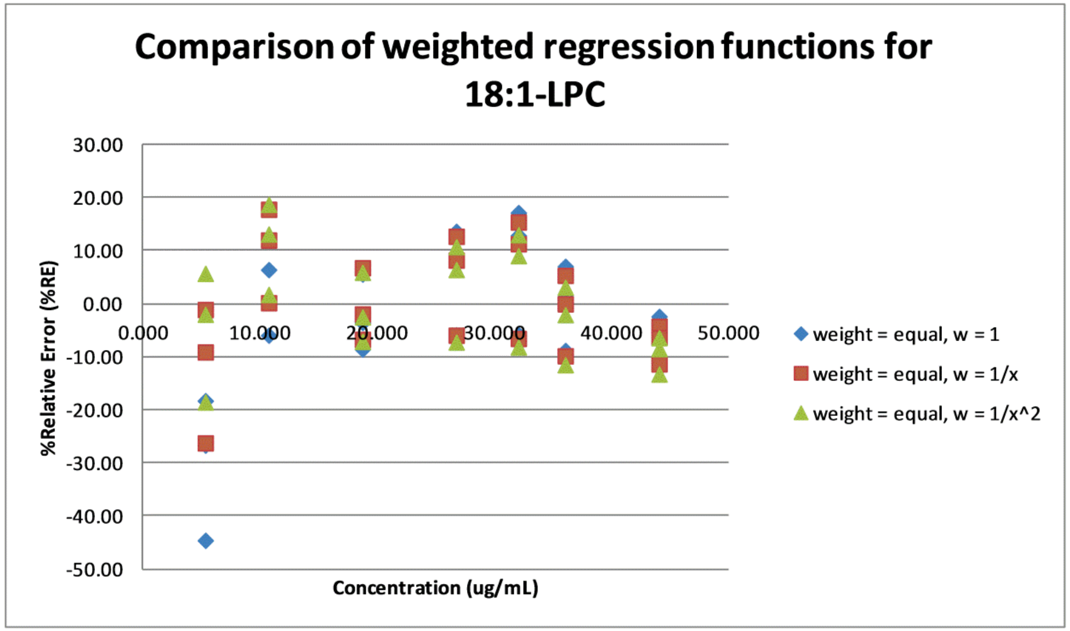

Fig. S2 Plot of the residual y-values versus concentration for 18:1-LPC at unweighted and 1/x weighted regression functions

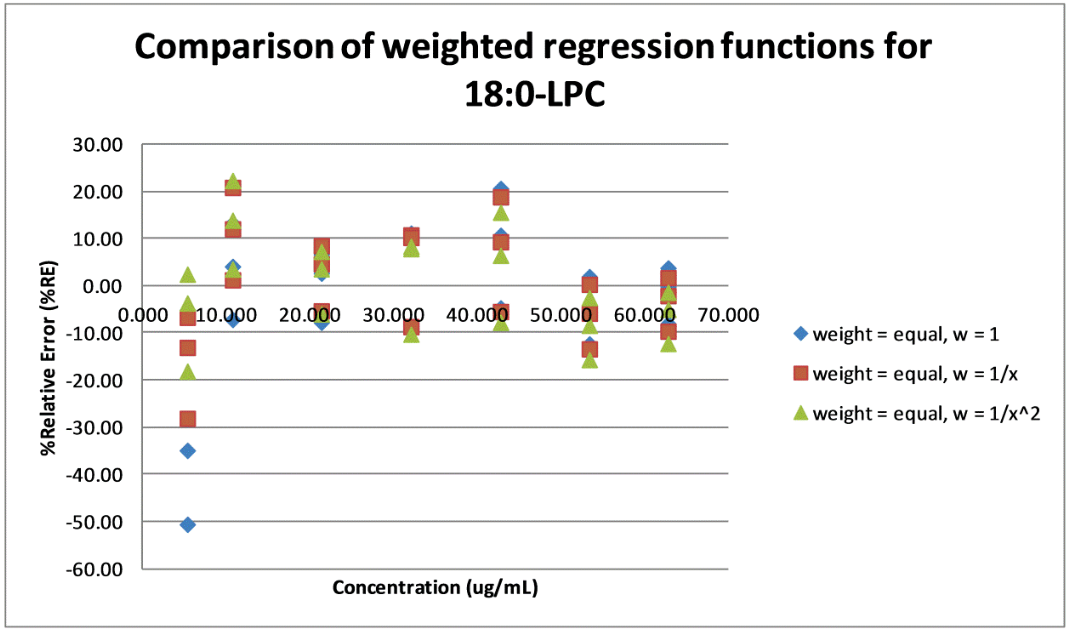

Fig. S3 Plot of the residual y-values versus concentration for 18:0-LPC at unweighted and 1/x weighted regression functions

Table S6 Data evaluating the homoscedasticity of the calibration standards

**Compound *(m/z)***	**Variance of Relative Response Factor**		**F-calc**	**F-stat (2,2, 0.95) one tailed**	**Homoscedastic?**
	**S1**	**S7**			
16:0-LPC (496.5)	0.0067	0.1453	21.65	19	No
18:1-LPC (522.5)	0.0298	0.1658	5.56	19	Yes
18:0-LPC (524.5)	0.0234	0.8999	38.51	19	No

Table S7 Percentage Relative Error (%RE) of the calibration standards for 16:0-LPC using unweighted and weighted linear regression. %RE = (calculated amount-true amount)/true amount * 100. The lower %RE indicates the most appropriate weighting function for the regression data

16:0-LPC					
Standard	Calculated concentration		%RE		
	weight = equal, w = 1	weight = equal, w = 1/x	weight = equal, w = 1	weight = equal, w = 1/x	weight = equal, w = 1/x^2
	y = 0.8459x + 1.2257	y = 0.9315x + 0.6610	y = 0.8459x + 1.2257	y = 0.9315x + 0.6610	y = 1.0111x + 0.4368
**S1**	0.13	4.18	-97.63	-21.19	0.62
**S1**	-0.21	3.87	-103.90	-26.90	-4.63
**S1**	-1.13	3.04	-121.26	-42.67	-19.15
**S2**	15.11	17.79	13.64	33.74	34.38
**S2**	12.43	15.35	-6.51	15.43	17.52
**S2**	11.22	14.25	-15.67	7.12	9.86
**S3**	24.76	26.55	-7.25	-0.56	-2.83
**S3**	23.31	25.23	-12.68	-5.49	-7.37
**S3**	22.11	24.14	-17.20	-9.59	-11.15
**S4**	46.50	46.29	24.68	24.11	18.32
**S4**	45.69	45.55	22.49	22.13	16.50
**S4**	37.59	38.20	0.77	2.40	-1.68
**S5**	66.12	64.10	27.15	23.27	16.43
**S5**	63.19	61.44	21.51	18.16	11.71
**S5**	53.69	52.82	3.25	1.58	-3.56
**S6**	77.26	74.22	3.42	-0.64	-6.48
**S6**	78.04	74.93	4.47	0.31	-5.60
**S6**	69.87	67.51	-6.47	-9.63	-14.75
**S7**	96.66	91.83	-3.34	-8.17	-13.91
**S7**	94.77	90.12	-5.23	-9.88	-15.49
**S7**	90.75	86.47	-9.25	-13.53	-18.85
**Sum %RE**			**-285.01%**	**-0.038%**	**-0.120%**

Table S8 Percentage Relative Error (%RE) of the calibration standards for 18:1-LPC using unweighted and weighted linear regression. %RE = (calculated amount-true amount)/true amount * 100. The lower %RE indicates the most appropriate weighting function for the regression data

18:1-LPC					
**Standard**	**Calculated concentration**		**%RE**		
	**weight = equal, w = 1**	**weight = equal, w = 1/x**	**weight = equal, w = 1**	**weight = equal, w = 1/x**	**weight = equal, w = 1/x^2**
	**y = 1.6248x + 1.1304**	**y = 1.7006x + 0.8503**	**y = 1.6248x + 1.1304**	**y = 1.7006x + 0.8503**	**y = 1.7632x + 0.7064**
**S1**	4.33	5.24	-18.29	-1.11	5.69
**S1**	3.89	4.82	-26.66	-9.11	-2.02
**S1**	2.94	3.91	-44.60	-26.24	-18.55
**S2**	12.03	12.60	12.47	17.77	18.70
**S2**	11.38	11.98	6.40	11.97	13.11
**S2**	10.06	10.72	-5.95	0.17	1.72
**S3**	19.74	19.97	5.57	6.77	5.90
**S3**	18.04	18.34	-3.55	-1.95	-2.50
**S3**	17.10	17.44	-8.56	-6.74	-7.13
**S4**	30.33	30.09	13.61	12.68	10.73
**S4**	29.08	28.89	8.93	8.21	6.41
**S4**	25.13	25.11	-5.89	-5.95	-7.24
**S5**	36.11	35.61	12.85	11.27	9.03
**S5**	37.49	36.92	17.15	15.38	12.99
**S5**	30.14	29.90	-5.82	-6.57	-8.18
**S6**	38.53	37.92	7.03	5.32	3.10
**S6**	36.51	35.98	1.41	-0.05	-2.08
**S6**	32.82	32.46	-8.83	-9.83	-11.51
**S7**	42.94	42.13	-2.41	-4.25	-6.41
**S7**	41.95	41.18	-4.67	-6.41	-8.49
**S7**	39.66	39.00	-9.86	-11.37	-13.27
**Sum %RE**			**-59.67%**	**-0.036%**	**0.017%**

Table S9 Percentage Relative Error (%RE) of the calibration standards for 18:0-LPC using unweighted and weighted linear regression. %RE = (calculated amount-true amount)/true amount * 100. The lower %RE indicates the most appropriate weighting function for the regression data

18:0-LPC					
**Standard**	**Calculated concentration**		**%RE**		
	**weight = equal, w = 1**	**weight = equal, w = 1/x**	**weight = equal, w = 1**	**weight = equal, w = 1/x**	**weight = equal, w = 1/x^2**
	**y = 1.6866x + 1.2367**	**y = 1.7564x + 0.8975**	**y = 1.6866x + 1.2367**	**y = 1.7564x + 0.8975**	**y = 1.8316x + 0.7097**
**S1**	3.45	4.61	-34.90	-13.08	-3.68
**S1**	3.80	4.94	-28.26	-6.70	2.44
**S1**	2.62	3.81	-50.58	-28.13	-18.11
**S2**	11.14	11.99	4.08	12.04	13.86
**S2**	12.12	12.93	13.23	20.82	22.28
**S2**	9.93	10.83	-7.16	1.24	3.51
**S3**	22.72	23.11	6.66	8.50	7.27
**S3**	21.86	22.29	2.65	4.64	3.57
**S3**	19.65	20.16	-7.75	-5.34	-6.00
**S4**	35.56	35.44	11.13	10.76	8.36
**S4**	35.38	35.27	10.57	10.22	7.85
**S4**	29.09	29.22	-9.10	-8.67	-10.27
**S5**	47.26	46.67	10.68	9.31	6.43
**S5**	51.49	50.74	20.59	18.83	15.56
**S5**	40.69	40.36	-4.72	-5.47	-7.75
**S6**	54.32	53.46	1.92	0.29	-2.54
**S6**	50.92	50.19	-4.46	-5.83	-8.41
**S6**	46.72	46.16	-12.34	-13.39	-15.66
**S7**	65.06	63.76	3.76	1.70	-1.38
**S7**	62.58	61.38	-0.20	-2.10	-5.02
**S7**	57.65	56.65	-8.05	-9.64	-12.26
**Sum %RE**			**-82.25%**	**-0.002%**	**0.066%**

Table S10 Regression statistics table for 1/x weighted regression for each LPC, including regression equation calculated using the concentration ratio, correlation coefficient (R) and coefficient of determination (R^2)

**Compound**	**Regression Equation**	**R**	**R^2**
16:0-LPC	y = 0.9315x + 0.6610	0.9848	0.9698
18:1-LPC	y = 1.7006x + 0.8503	0.9891	0.9783
18:0-LPC	y = 1.7564x + 0.8975	0.9909	0.9819

Table S11 Individual quality control data showing calculated concentration in µg/mL, percentage accuracy and precision (represented as a %CV) for five replicate samples at two concentrations within the calibration range

**Compound**	**Calculated Concentration (µg/mL)**		**True Concentration (µg/mL)**		**Accuracy (%)**		**Mean Accuracy (%)**		**Precision (%CV)**	
	**QC1**	**QC2**	**QC1**	**QC2**	**QC1**	**QC2**	**QC1**	**QC2**	**QC1**	**QC2**
**16:0-LPC**	43.71	66.29	37.30	74.70	17.18	-11.26	7.34	-8.03	9.34	4.09
	42.61	65.28			14.24	-12.61				
	36.06	69.44			-3.32	-7.04				
	35.95	71.77			-3.62	-3.93				
	41.86	70.75			12.23	-5.29				
**18:1-LPC**	27.88	31.07	26.70	36.00	4.42	-13.71	-5.66	-11.14	8.19	3.57
	26.96	30.52			0.97	-15.22				
	23.59	32.72			-11.63	-9.12				
	23.79	32.44			-10.91	-9.88				
	23.72	33.20			-11.15	-7.78				
**18:0-LPC**	37.36	51.37	32.00	53.30	16.75	-3.62	5.24	-3.84	10.20	4.60
	37.45	47.21			17.03	-11.42				
	31.27	52.05			-2.28	-2.35				
	30.50	52.38			-4.70	-1.73				
	31.81	53.26			-0.60	-0.08				

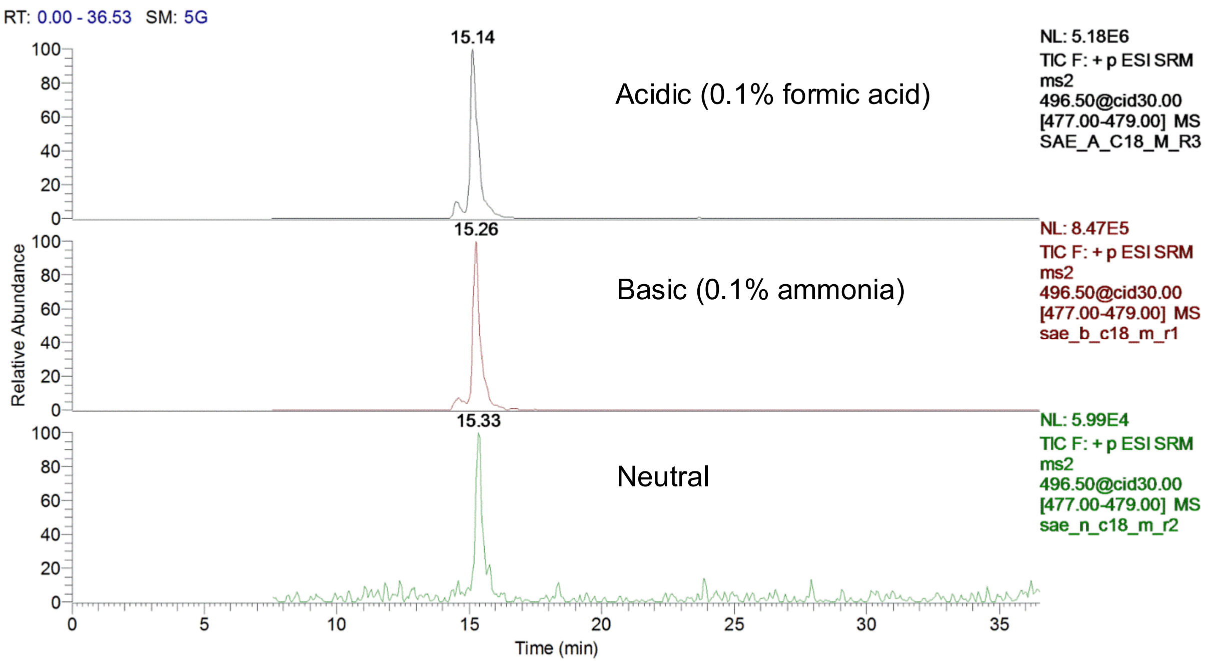

Fig. S4 Extracted ion chromatograms for 16:0-LPC of plasma samples prepared using solid phase extraction (SPE) under acidic, basic and neutral conditions. Quantities were determined using a calibration line run with the plasma samples. Regression statistics obtained included the regression equation of $y = 0.5207x + 0.2730$ and $R^2 = 0.9922$

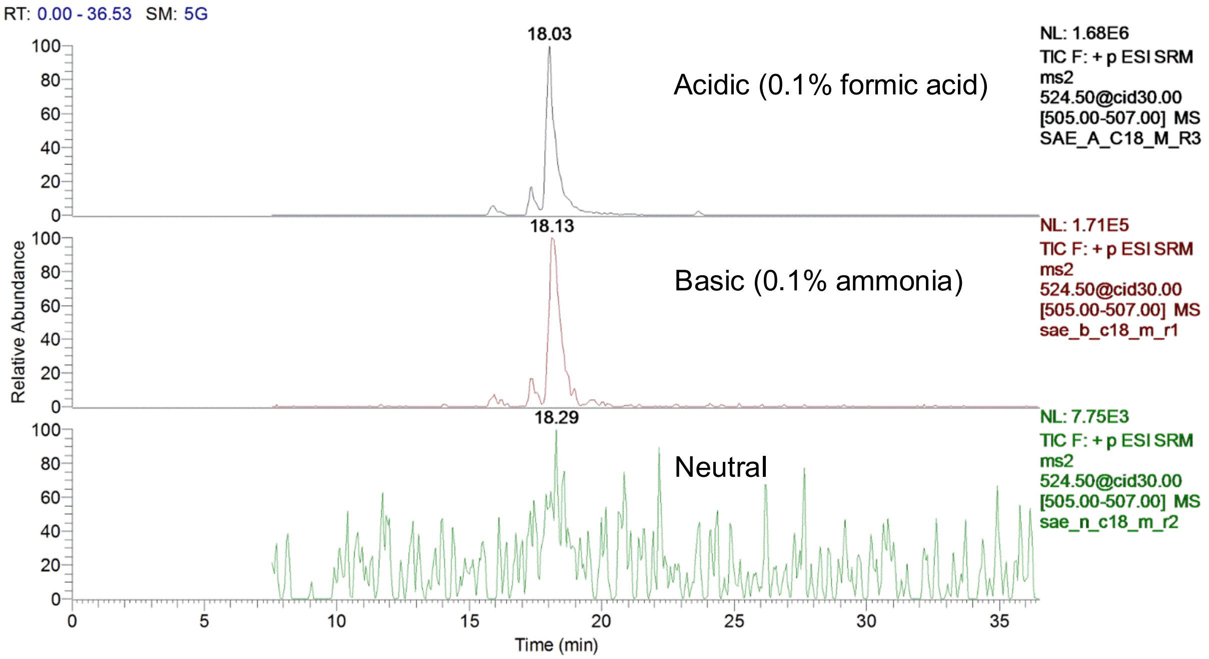

Fig. S5 Extracted ion chromatograms for 18:0-LPC of plasma samples prepared using solid phase extraction (SPE) under acidic, basic and neutral conditions. Quantities were determined using a calibration line run with the plasma samples. Regression statistics obtained included the regression equation of $y = 0.9271x + 0.1976$ and $R^2 = 0.9989$
